# ALK5 inhibitor impact on bleomycin-induced systemic sclerosis mouse model via multifunctional optical coherence tomography

**DOI:** 10.1063/5.0281447

**Published:** 2025-11-06

**Authors:** Pavel V. Nikitin, Harshdeep S. Chawla, Jessica Gutierrez, Geethangili Madamanchi, Manmohan Singh, Salavat R. Aglyamov, Minghua Wu, Jerry Alonso, Matthew Fronheiser, Juliana Coculo, Shuyan Du, Shervin Assassi, Kirill V. Larin

**Affiliations:** 1 Department of Biomedical Engineering, University of Houston, 3517 Cullen Blvd, Houston, Texas 77204, USA; 2 Division of Rheumatology, Department of Internal Medicine, University of Texas Health Science Center at Houston, McGovern Medical School, 6431 Fannin, Houston, Texas 77030, USA; 3 Bristol Myers Squibb, Route 206 & Province Line Road, Princeton, New Jersey 08543, USA

## Abstract

Systemic sclerosis (SSc) is a chronic autoimmune disease characterized by fibrosis, vascular dysfunction, and immune dysregulation, leading to significant morbidity and mortality. Noninvasive imaging techniques are critical for monitoring disease progression and evaluating therapeutic interventions. This study investigates the technical feasibility of multifunctional optical coherence tomography (OCT)-based methods for longitudinal assessment of skin thickness, stiffness, and microvasculature in a murine SSc model as a translational, noninvasive, and quantitative method to study disease progression and treatment response. Our findings demonstrate significant structural, biomechanical, and vascular changes in the skin's stiffness, indicative of fibrosis, a hallmark of SSc. The application of SB 525334 (a transforming growth factor β1 receptor ALK5 inhibitor) mitigated these changes, highlighting its potential as a treatment strategy. Despite the inherent limitations of the mouse model in replicating the complexity of SSc, this study introduces a new technique for investigating the SSc pathogenesis and evaluating the efficacy of potential SSc therapies. These results encourage further exploration of the multifunctional Optical Coherence Elastography and OCT Angiography for monitoring disease progression and treatment response in SSc. In summary, bleomycin treatment significantly increased skin thickness, stiffness, and vessel lumen width, while SB 525334 partially reversed these changes, demonstrating the feasibility of our multifunctional OCT approach for monitoring experimental SSc.

## INTRODUCTION

Changes in tissue biomechanical properties are well-known to correlate with many pathological processes.^1,2^ Typically, changes in the structural, elastic, and viscous properties of various organs and body parts are perceived as degeneration of tissue components, leading to progressing pathological states, such as osteoarthritis or chronic heart failure.^3,4^ For example, disruptions in the biomechanical properties of the connective tissue framework that, although rare, can lead to irreversible damage to vital structures of the organism, causing disability and even death, often at a relatively young age.^5–8^ One such disease is systemic sclerosis (SSc), an autoimmune disease that predominantly affects the connective tissue of the skin, lungs, esophagus, blood vessels, stomach, heart, and kidneys.^9,10^ SSc is a systemic condition that can lead to interstitial lung disease, pulmonary hypertension, and gastrointestinal dysmotility, and current imaging modalities such as ultrasound and high-resolution X-ray computed tomography (CT) are being explored for multi-organ assessment.^11^

Chronic inflammation with recurrent exacerbations in SSc patients leads to remodeling of the connective tissue framework, which underlies the profound functional reorganization of these tissues.^12,13^ The basis of connective tissue remodeling is the activation of autologous clones of T- and B-lymphocytes as well as NK cells, resulting in damage to the normal network of collagen and elastin fibers, culminating in pathological fibrosis. This biomechanical rearrangement, which is characterized by a significant increase in tissue stiffness, inevitably affects the physiological activity of the affected tissues and organs, causing rapidly progressing dysfunction.^14–17^ The skin, particularly in the distal part of the upper limbs and on the face, is most frequently affected, exhibiting typical signs of SSc—increased skin stiffness, development of sclerodactyly, and trophic disturbances.^18,19^

The predominant affliction of SSc in the skin makes it feasible to use dermally focused models, where skin and subcutaneous structures undergo fibrotic changes, to study the mechanisms of disease development and potential therapies.^20,21^ The potential for evaluating novel treatments is particularly important as there are limited approved disease-modifying agents for SSc.^22,23^ The discovery of a targeted therapeutic solution is complicated by the complexity of SSc pathogenesis, which is composed of an autoimmune process, pronounced fibrosis, and vascular lesions. Ideally, a potential therapeutic target should affect all parts of the pathological process, which, considering this multidimensionality, is quite challenging. In this context, the transforming growth factor β1 (TGF-β1) signaling pathway deserves special attention. The TGF-β1 receptor ALK5 mediates signaling that is crucial for fibroblast differentiation into myofibroblasts, thus regulating collagen production and fibrosis. Additionally, TGF-β/ALK5 signaling contributes to vascular dysfunction and immune system dysregulation, further exacerbating SSc manifestations.^24–27^ Therefore, we investigated targeting ALK5 as a therapeutic strategy, which we evaluated longitudinally with optical and biomechanical imaging.

In this study, SSc-like changes in the skin were induced by the profibrotic agent bleomycin in a murine model.^28,29^ Noninvasive and longitudinal assessment of structural changes in the skin under the influence of bleomycin alone and in combination with the selective ALK5 inhibitor SB 525334 was performed using optical coherence tomography (OCT)^30^ to image the structural changes in the skin. Although ultrasound,^9^ magnetic resonance,^31,32^ and x-ray CT imaging^33^ have been utilized for characterizing dermal manifestations of SSc, they lack the spatial resolution and contrast for visualizing minute changes in the skin structure, such as the disappearance of the epidermis–dermis junction, which is a hallmark of SSc.^34^ The micrometer-scale resolution, intrinsic optical contrast, and wide dynamic range make OCT particularly well-suited for imaging fine structures in mouse skin.

In addition to structural imaging, we utilized the elastographic functional extension of OCT, termed optical coherence elastography (OCE),^35^ to assess the changes in skin biomechanical properties associated with fibrosis and the effects of the potential therapeutic effects of SB 525334. OCE has proven to be effective in evaluating the viscoelastic consequences of pathologic changes in various tissues,^36,37^ including fibrosis in the skin.^38,39^ Similar to structural imaging with OCT, OCE carries many of the same benefits, such as improved resolution and contrast. Moreover, OCT structural imaging comes free with OCE imaging, i.e., an OCT structural image is generated during OCE imaging, easily enabling multifunctional imaging.

SSc-associated fibrosis can also significantly affect microvasculature.^40,41^ Currently, physicians utilize a simple camera and microscope to visualize changes in the microvasculature of the nailfold in the fingers, i.e., nailfold capillaroscopy, for SSc diagnosis.^42,43^ However, this method is highly qualitative and cannot provide a three-dimensional mapping of the microvasculature. Thus, the combination of skin thickness assessment using structural OCT, mechanical characterization with OCE, and microvasculature imaging with OCT angiography (OCTA) enables rigorous and multifunctional assessment of the impact of SB 525334 on this pathological process.^44,45^

## RESULTS

The experiment was conducted in two phases, dividing the total population of mice into two equal batches (30 animals per phase), using an identical experimental design for each phase. This fractionation was applied to ensure the experimenters could perform all experimental procedures with high quality. Where statistical significance coincided with small absolute differences, we report these as modest effects and interpret them cautiously with respect to biological relevance.

### Skin thickness measurements

**FIG. 1.**
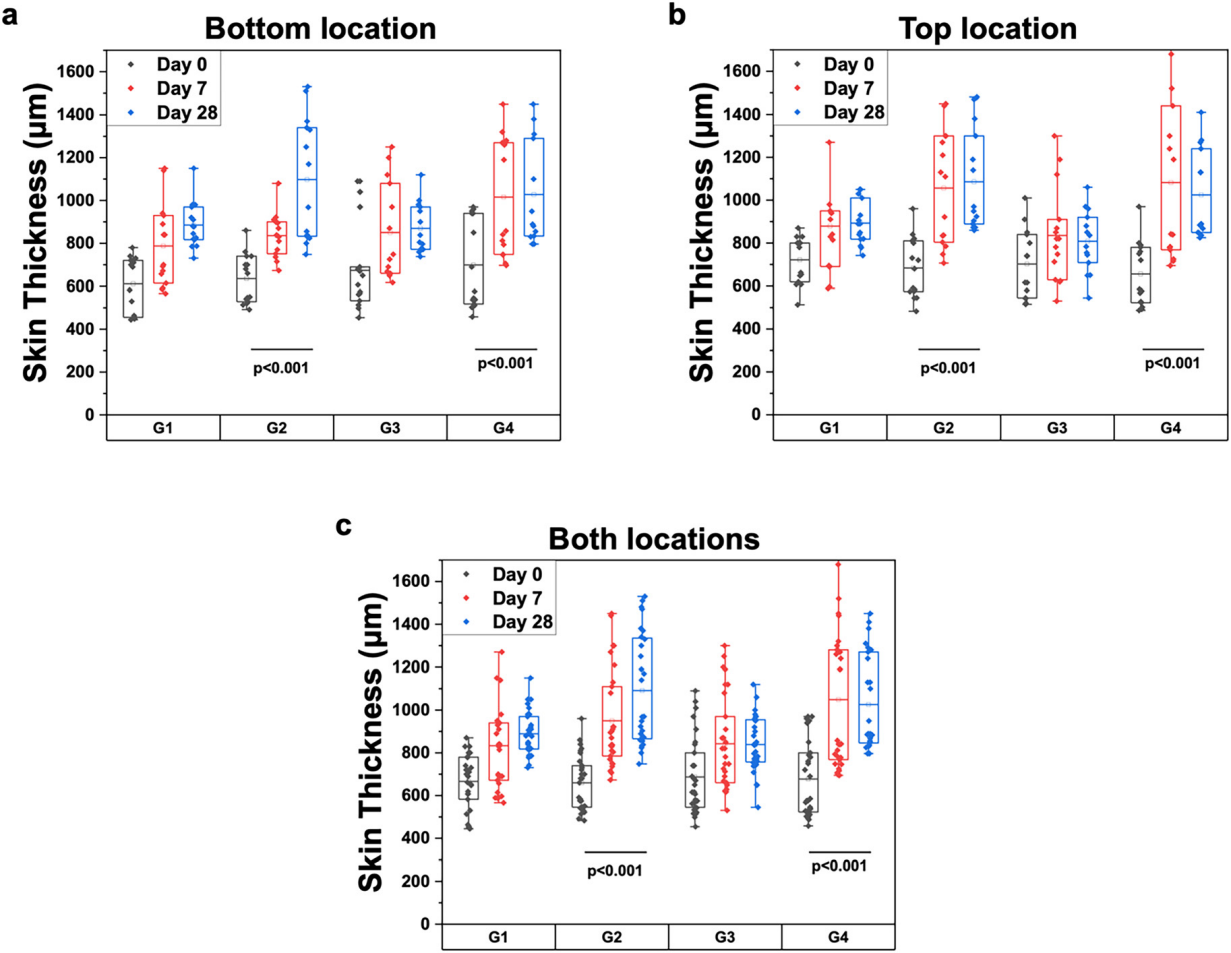
Comparative skin thickness measurements in all study groups. Skin thickness as assessed by OCT structural imaging at the (a) bottom site, (b) top site, and (c) average from both sites. G1: mice administered saline + vehicle. G2: mice administered bleomycin + vehicle. G3: mice administered saline + SB525334. G4: mice administered bleomycin + SB525334.

Comparative measurements of skin thickness were conducted using OCT structural imaging across all study groups. The results in Fig. 1 demonstrate a statistically significant increase in skin thickness after 28 days both for the group that received only bleomycin injections (group 2, p < 0.001; pooled across both sites: median Δ = 406 *μ*m, 95% CI [324, 525] *μ*m) and for the group treated with a combination of bleomycin injections and SB 525334 gavage (group 4, p < 0.001; pooled across both sites: median Δ = 361 *μ*m, 95% CI [327, 425] *μ*m).

### Biomechanical skin response to bleomycin

**FIG. 2.**
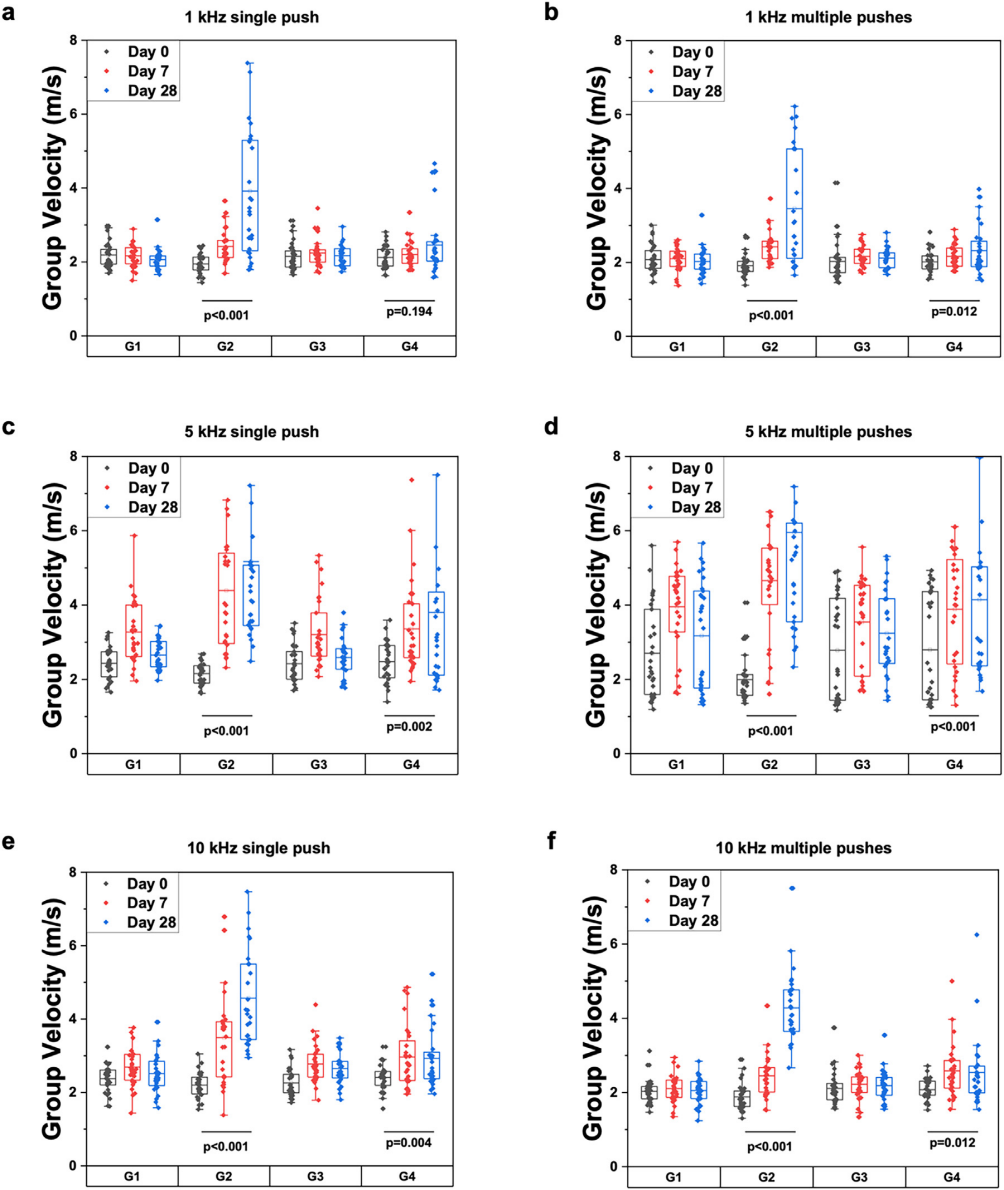
Group velocity measurements at both locations. Average group velocity at both locations for each study group over time and at each wave excitation scheme: (a) 1 kHz with a single push, (b) 1 kHz with multiple pushes, (c) 5 kHz with a single push, (d) 5 kHz with multiple pushes, (e) 10 kHz with a single push, and (f) 10 kHz with multiple pushes. G1: mice administered saline + vehicle. G2: mice administered bleomycin + vehicle. G3: mice administered saline + SB525334. G4: mice administered bleomycin + SB525334.

Since bleomycin induces dermal fibrosis similar to the pathogenetic factors of SSc,^28^ an expected effect of its administration is an increase in skin stiffness. The results we obtained reflect the expected stiffening due to fibrosis—across all frequencies of mechanical excitation used (1, 5, and 10 kHz), as well as with single or multiple pushes. The skin of the mice injected with bleomycin without SB 525334 (group 2) became significantly stiffer after 4 weeks (p < 0.001 for all frequencies and pushes, pooled both sites: median Δ 1.630 m/s, 95% CI [0.600, 3.012] m/s; Fig. 2, supplementary material Figs. 1 and 2). Both control groups (Groups 1 and 3) showed a slight increase in skin stiffness on day 7, which was not statistically significant (G1: p = 0.546; pooled both sites median Δ −0.098 m/s, 95% CI [−0.178, 0.139] m/s; G3: p = 0.075; pooled both sites median Δ 0.065 m/s, 95% CI [−0.095, 0.333] m/s) from the start of the experiment, possibly due to the response to the injections (Fig. 2, supplementary material Figs. 1 and 2). However, the magnitude of stiffening varied across excitation frequencies and push modes. Different frequencies exhibited distinct sensitivity to fibrosis, likely reflecting depth-dependent heterogeneity in mechanical properties and the influence of skin viscoelasticity. Because elastic-wave propagation is frequency-dependent, lower excitation (1 kHz) interrogates deeper dermal layers, whereas higher frequencies (5 and 10 kHz) are more sensitive to the superficial regions. Moreover, higher excitation frequencies exhibit greater sensitivity to the viscoelastic components of the skin mechanical response.

### Impact of SB 525334 treatment

Convinced of the effectiveness of the experimental model, we focused on assessing the results of SB 525334 administration in conjunction with bleomycin administration (group 4) on skin fibrosis. The trends identified here were heterogeneous, where the statistical significance varied at different mechanical wave frequencies. However, it is important to compare the impact of SB 525334 on fibrosis progression in group 4 to the untreated fibrosis in group 2. For example, while certain excitation parameters such as 1 kHz, 1 push (top and bottom locations), and 10 kHz, 1 push (bottom site only, supplementary material Fig. 1) as well as 10 kHz, three pushes (top site only, supplementary material Fig. 2) showed no significant differences in skin stiffness of mice in group 4 between baseline and day 28 (p = 0.068, p = 0.052, p = 0.542, respectively), this was not the case in group 2, which received bleomycin injections without SB 525334 administration (Table I).

**TABLE I.** P-values from the Wilcoxon Signed Rank test to assess the significance of changes in the group velocity of mechanical waves under varying excitation parameters between day 0 and 4 weeks (day 28) across all study groups.

Mechanical excitation parameters	Group 1 (saline + vehicle)			Group 2 (bleo + vehicle)			Group 3 (saline + SB525334)			Group 4 (bleo + SB525334)		
	Top	Bottom	Both	Top	Bottom	Both	Top	Bottom	Both	Top	Bottom	Both
1 kHz 1 push	0.106	0.293	0.084	<0.001	<0.001	<0.001	0.952	0.194	0.250	0.754	0.068	0.194
1 kHz 3 pushes	0.804	0.489	0.472	<0.001	<0.001	<0.001	0.241	0.052	0.089	0.124	0.008	0.002
5 kHz 1 push	0.524	0.118	0.201	<0.001	<0.001	<0.001	0.626	0.127	0.063	0.286	0.014	0.001
5 kHz 3 pushes	0.389	0.121	0.077	<0.001	<0.001	<0.001	0.059	0.180	0.102	0.001	0.008	<0.001
10 kHz 1 push	0.234	0.118	0.256	<0.001	<0.001	<0.001	0.057	0.994	0.998	0.025	0.052	0.004
10 kHz 3 pushes	0.998	0.268	0.382	<0.001	<0.001	<0.001	0.318	0.391	0.162	0.542	0.008	0.012

When aggregating results from both imaging locations, only the data for 1 kHz, single push demonstrated no significant differences between day 1 and day 28 in group 4 (p = 0.194). Despite this, the overall effect of SB 525334 was evident when comparing group 4 to group 2. The increase in skin stiffness between day 0 and day 28 in group 4 was substantially lower compared to group 2 across most excitation parameters, indicating that SB 525334 ameliorated the fibrotic impact of bleomycin. A direct statistical comparison of elastic-wave group velocity on day 28 between group 2 and group 4 showed significant differences in all cases (Table II; pooled both sites: HL median difference G4–G2 −1.312 m/s, 95% CI [−2.612, −0.181] m/s). This analysis demonstrates that SB 525334 was effective in reducing the progression of bleomycin-induced fibrosis, as the group treated with SB 525334 exhibited significantly less stiff skin as compared to the untreated group (Fig. 2, supplementary material Figs. 1 and 2).

**TABLE II.** P-values from the Mann–Whitney U-test to assess the significance of differences in the group velocity of mechanical waves under varying excitation parameters on day 28 between group 2 (Bleo + Vehicle) and group 4 (Bleo + SB525334).

1 kHz 1 push	1 kHz 3 pushes	5 kHz 1 push	5 kHz 3 pushes	10 kHz 1 push	10 kHz 3 pushes
0.001	0.008	0.007	0.010	<0.001	<0.001

### Microvasculature changes

**FIG. 3.**
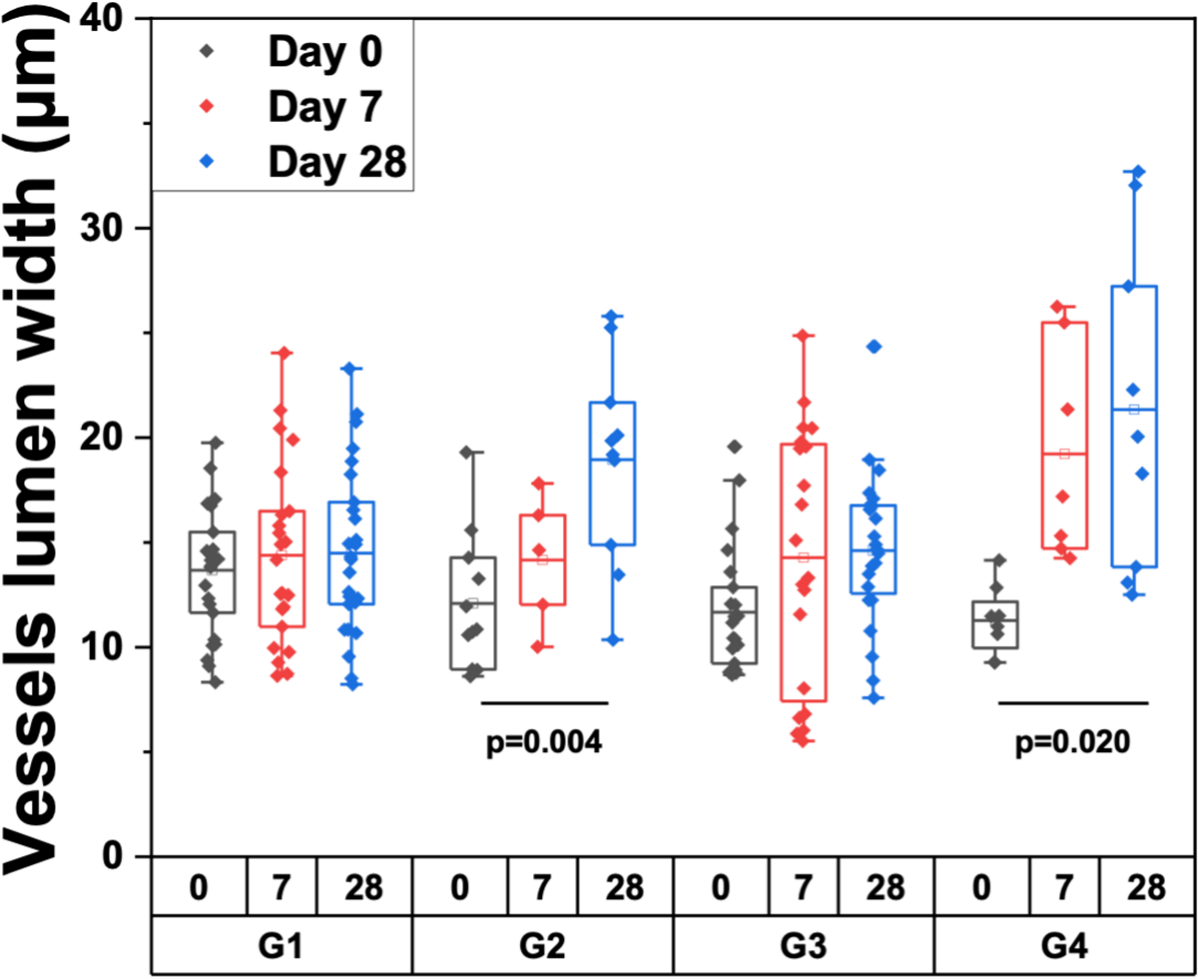
Vessels lumen width measurements. Vessel lumen width measurements based on OCTA at both sites were averaged. G1: mice administered saline + vehicle. G2: mice administered bleomycin + vehicle. G3: mice administered saline + SB525334. G4: mice administered bleomycin + SB525334.

We did not expect the bleomycin dermal fibrosis model to exhibit vasculopathy changes characteristic of SSc. However, from the OCTA images, we observed an increased vessel lumen diameter after bleomycin injection with or without SB 525334 treatment, which is most likely due to fibrosis and the resulting angioectasia in the surrounding tissue. A comparison of vessel lumen width between group 2 animals on day 0 and day 28 showed a significant increase as a result of bleomycin (p = 0.004; pooled across both sites: median Δ = 8.08 *μ*m, 95% CI [2.86, 12.75] *μ*m). Similarly, significant angioectasia was also observed in group 4 (p = 0.020; pooled across both sites: median Δ = 6.70 *μ*m, 95% CI [4.16, 16.82] *μ*m), but its severity was reduced when compared with group 2. This vascular data suggest that while SB 525334 helps mitigate the pathological changes associated with fibrosis, including angioectasia, it does not fully restore vessel lumen diameter to control levels. Ideally, a vessel diameter closer to that of the control group would indicate a more complete reversal of the fibrotic effects. Therefore, although SB 525334 reduces the severity of angioectasia, the fact that the median vessel lumen remains wider than in the control group indicates that the therapeutic effect is partial, and that further optimization or additional treatments may be necessary to achieve more normal vessel characteristics (Fig. 3, supplementary material Fig. 3).

### Histology results

**FIG. 4.**
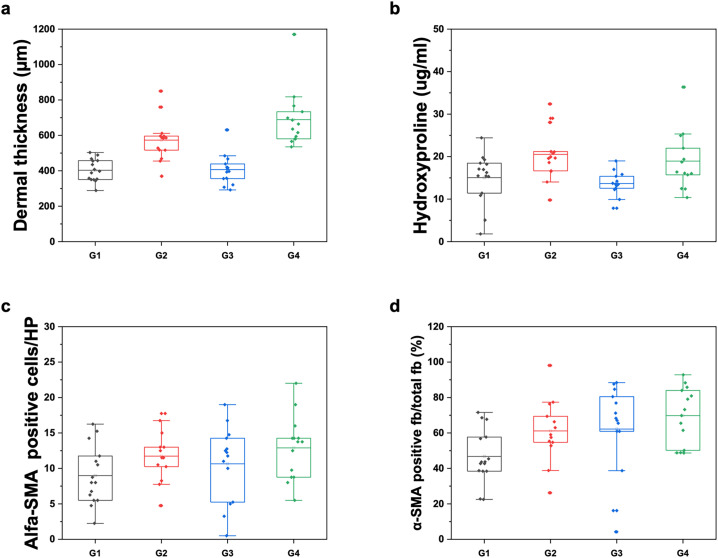
Histological analysis of skin samples at day 28 from different experimental groups. The histological analysis, including (a) dermal thickness, (b) hydroxyproline, (c) α-smooth muscle actin (α-SMA) immunohistochemistry analysis of the α-SMA positive fibroblasts number per HP, and (d) ratio of α-SMA positive fibroblasts to total fibroblasts number. n = 15 per group.

The histopathological analysis, paralleling our OCT measurements, demonstrated that bleomycin-injected mice (Groups 2 and 4) exhibited significantly increased dermal thickness and hydroxyproline levels compared to the control groups (Groups 1 and 3), confirming the efficacy of the SSc-associated fibrosis model. Specifically, Masson's Trichrome staining results indicated a clear increase in dermal thickness in bleomycin-treated groups, as shown in Fig. 4 (dermal thickness: p < 0.001; HL median difference = 208 *μ*m, 95% CI [162, 259] *μ*m; hydroxyproline: p = 0.0009; HL median difference = 4.35 *μ*g/ml, 95% CI [2.00, 7.33] *μ*g/ml).

## DISCUSSION

In this work, we utilized multifunctional OCT, comprising OCT, OCE, and OCTA to assess the longitudinal changes in tissue structure, stiffness, and microvasculature, respectively, in bleomycin-induced fibrosis in the skin of mice over time. The bleomycin-induced skin fibrosis model served as an analog to the fibrotic changes in the skin associated with SSc.

While no animal model reproduces the full spectrum of SSc, the bleomycin-induced dermal fibrosis model is widely used and well characterized for robust skin involvement, and our conclusions are limited to these aspects of disease. Bleomycin predictably affected the biomechanical properties of the skin, causing a significant increase in its stiffness, as evidenced by the substantial rise in group velocity (i.e., stiffening) in the skin of the bleomycin-treated group. Notably, the increase in skin stiffness was observed as early as one week after the onset of injections, which may be associated with reactive inflammation induced by the injection's physical injury to the skin.^46,47^ Even though our imaging was not at the injection site, the localized insult from the injection was still significant enough to affect nearby imaged regions. This reactive process, as indicated by the results in saline + vehicle gavage treated group (group 1) and saline + SB 525334 treated group (group 3), is virtually neutralized by day 28, thus not significantly impacting the outcome of the statistical results comparing the stiffnesses from day 0 to day 28. This conclusion is confirmed by literature data obtained on a similar bleomycin model.^29,38^ However, it is interesting to note that considering this side effect from bleomycin administration as purely undesirable is a little more nuanced. This is because mechanical stress and minor injury induced by the injection process can simulate the inflammatory responses and subsequent fibrotic processes characteristic of SSc. Nonetheless, inflammation, both related to injections and primarily caused by bleomycin, is not always beneficial for the model used. It is precisely this inflammation that maintains a state of intratissue edema, which significantly complicates the determination of skin thickness, along with the skin pigmentation caused by the injections and hair removal. Despite the mixed results of this part of the study, they largely support the validity of the model used, especially in the context of the other parameters studied.

Having examined the model and its characteristics, we also considered the effectiveness of the selective ALK5 inhibitor within this context. Although the results are heterogeneous, they, nonetheless, suggest that the drug exerts a beneficial effect. The frequency dependence of the OCE results is expected from depth-weighted viscoelastic sampling: higher-frequency (10 kHz) waves are more confined to superficial layers, whereas 1 kHz interrogates deeper dermal–subcutaneous tissue. SB-525334 produced the greatest mitigation at 10 kHz, with weaker effects at 1 kHz, consistent with earlier, superficial mechanical improvement (e.g., reduced myofibroblast tone/edema and reorganization of upper-dermal matrix) preceding deeper changes over a 28-day window. Our histology was bulk/section-based and not layer-resolved; thus, it is not optimized to mirror frequency-weighted mechanics on a layer-by-layer basis within the same animal. This explains the heterogeneous OCE responses without implying inconsistency, and we avoid over-interpretation pending layer-resolved histology in future work. Comparing group 4 (bleomycin + SB-525334) to group 2 (bleomycin only), we observed that SB-525334 significantly reduced the progression of OCE-derived skin stiffening, indicating biomechanical improvement *in vivo* despite minimal change in the histologic endpoints over 28 days.^24–26,48,49^ The finding that SB-525334 reduced OCE elastic-wave velocity while histologic markers (dermal thickness, hydroxyproline, α-SMA) changed minimally over the same 28-day window is biologically plausible. OCE captures an *in vivo*, layer-weighted viscoelastic response influenced by matrix organization (fiber alignment/crimp), cross-linking state, interstitial fluid/edema, and myofibroblast contractile tone—features that can improve early with TGF-β pathway inhibition. Bulk histology, by contrast, is mass/area-based, section-limited, and terminal. It preferentially reflects later, larger reductions in collagen burden or compartment thickness and can lag behind earlier mechanical/architectural remodeling. Thus, the current data are consistent with early biomechanical mitigation without definitive histologic reversal at day 28.

These OCE findings are consistent with our prior multifunctional OCT work in the same bleomycin model, which likewise showed elevated elastic-wave speeds in fibrotic vs control skin by week 4 and higher speeds in untreated fibrotic skin relative to a treatment cohort, with treated values intermediate and not significantly different from the fibrotic group (p = 0.18). Differences in effect magnitude across studies likely reflect distinct excitation schemes and therapeutics (imatinib previously vs ALK5 inhibition here), sample sizes, and boundary conditions, but together they support OCE as a sensitive readout of fibrosis-induced stiffening and its partial attenuation under anti-fibrotic intervention.^49^ Regarding the microvasculature, OCTA imaging revealed significant changes, supporting the beneficial effects of SB 525334. Bleomycin increased the perfused-lumen width in the imaged plexus. In the context of cutaneous fibrosis, such apparent dilation is plausible for several reasons. First, endothelial injury and inflammation can produce hyperemia with reduced vasomotor tone. Second, perivascular matrix remodeling and edema can lower extramural constraint, favoring outward (positive) remodeling of flow-bearing channels. Finally, microvascular rarefaction can prune small branches, shifting flow into fewer, larger paths and increasing the OCTA-measured lumen of vessels that remain perfused. Under SB-525334, the lumen-width increase was not attenuated, consistent with partial normalization of tone and matrix mechanics rather than frank vasoconstriction. Importantly, OCTA images blood flow, so it can only capture the perfused lumen, not wall thickness. It does not distinguish outward remodeling from true vasodilation, so we avoid mechanistic over-interpretation.^49^ Overall, these results suggest that SB 525334 cannot completely reverse the fibrotic changes in skin and subcutaneous tissue, but it can significantly slow the progression of SSc by reducing skin stiffness.

While our study demonstrates the potential of using OCT, OCE, and OCTA to assess fibrotic changes, it is essential to acknowledge the limitations of these imaging modalities. For instance, although OCT provides high-resolution structural images, its axial and lateral resolutions are limited when imaging small animals such as mice. A higher resolution system might be necessary to capture finer details of the mouse skin, which are crucial for a more accurate assessment of fibrosis progression. Moreover, OCTA is prone to motion artifacts, which can affect the accuracy of microvasculature imaging. These artifacts, along with the potential for noise in the OCE measurements, can complicate data interpretation. Future studies may benefit from integrating mechanical quantitation methods, such as a layered skin model, to better capture the mechanical properties of different skin layers and improve the robustness of the measurements.^36,50^

Despite these limitations, the multifunctional nature of the OCT system used in this study offers significant advantages. The ability to obtain structural, biomechanical, and microvascular data using a single system allows for a comprehensive assessment of the skin's condition. This approach not only enhances the efficiency of data collection but also enables the simultaneous analysis of multiple parameters, which is particularly beneficial in longitudinal studies like ours. Furthermore, while the current system is well-suited for deep imaging in human tissues, optimizing the resolution for smaller animal models could provide more detailed insights into disease progression and treatment effects. Because human clinical scoring instruments (e.g., the modified Rodnan skin score and related bedside scales) are not applicable in mice, we report quantitative OCT/OCE/OCTA metrics (thickness, stiffness, and vascularity) as murine surrogates. As a next step, we will pursue cross-modality validation alongside OCT (e.g., high-frequency ultrasound elastography and complementary angiographic imaging) to derive bridging relationships to clinical endpoints.

Although findings in mice do not directly translate to human SSc, there is currently no established minimal clinically important difference for OCT/OCE/OCTA metrics. As a pragmatic preclinical anchor, a change likely to warrant clinical follow-up would achieve at least a moderate standardized effect on the primary mechanical end point (Hedges' g ≳ 0.5), corresponding to a ∼15% to 20% relative change in OCE elastic-wave velocity vs fibrotic control, and occur without worsening of structural thickness or microvascular metrics across time points. In this study, the mechanical signal meets these preclinical criteria for biomechanical mitigation, while histologic reversal was not demonstrated within 28 days.

The choice of single-push and multiple-push excitation methods in OCE was intentional to capture different aspects of the skin's biomechanical response. Single-push excitations are useful for assessing low frequency elastic properties and viscoelastic properties by analyzing wave dispersion generally. In contrast, multiple push excitations allow for more depth-wise tailored analysis at each corresponding elastic wave wavelength. The combination of these methods provides a more comprehensive understanding of the biomechanical changes occurring in fibrotic skin, particularly in response to therapeutic interventions.

The literature, albeit limited, largely aligns with our findings. For instance, Fujiwara *et al.* demonstrated that MFG-E8 reduces fibrosis in SSc by inhibiting TGF-β signaling, thereby decreasing collagen production and fibrotic markers, which underscores its therapeutic potential against the disease.^51^ In another study, Terashima *et al.* showed that the TGF-β type I receptor inhibitor R-268712 prevents glomerular sclerosis, providing insights into the potential of ALK5 inhibitors to curb fibrosis in SSc.^52^ Thus, our research contributes additional insight into the fundamental mechanisms underlying the development of SSc symptoms and the role of TGF-β signaling in manifesting skin fibrosis and, at the same time, demonstrates the promising therapeutic potential of the TGF-β1 receptor ALK5 inhibitor SB 525334 for fibrosis associated with SSc. Our work also shows that a multifunctional approach to imaging the fibrotic effects in skin with OCT, OCE, and OCTA can provide a robust view of many different facets of disease progression and therapy efficacy.

This study undoubtedly has certain limitations. Despite the close resemblance of the bleomycin mouse model to the actual disease pathology in SSc, it cannot fully replicate the pathological mechanisms of SSc in all their complexity, though it does allow for the simulation of key pathological and symptomatic mechanisms and manifestations. Another limitation of this study is the lack of formal blinding during treatment administration and outcome assessment, which can introduce observer bias; to mitigate this risk, we used standardized acquisition settings and the deterministic, pre-specified analysis code for all imaging endpoints. A further limitation is the absence of a benchmark anti-fibrotic positive control (e.g., nintedanib or pirfenidone). We selected the ALK5 inhibitor SB-525334 as a mechanistic probe of TGF-β pathway modulation rather than as a head-to-head comparator and prioritized longitudinal, within-animal imaging across multiple endpoints (OCT/OCE/OCTA) to mitigate interpretive risk. Future work will incorporate a dedicated positive-control arm to benchmark effect sizes and strengthen assay validity across structural, biomechanical, and microvascular readouts as our recent work demonstrated the sensitivity of OCE to imatinib.^49^ Finally, a limitation is the partial discordance between OCE and histology at 28 days. Because OCE is sensitive to early changes in matrix organization, cross-linking, edema, and contractile tone, mechanical improvement may precede bulk histologic reversal, and layer-resolved histology will be needed for alignment.

## CONCLUSION

Within this study, we integrated structural, biomechanical, and angiographic optical coherence tomography (OCT, OCE, and OCTA) to longitudinally quantify skin fibrosis and vascular changes in a bleomycin-induced mouse model of systemic sclerosis. To our knowledge, this is the first application of such a multimodal OCT platform for continuous, noninvasive monitoring of fibrotic progression and therapeutic response *in vivo*. The ability to capture synchronized changes in tissue thickness, stiffness, and microvascular morphology demonstrates the power of multifunctional OCT to provide a comprehensive, quantitative assessment of fibrotic skin disease. Evaluating the ALK5 inhibitor SB 525334 within this framework revealed that targeted blockade of TGF-β signaling can partially slow fibrosis while emphasizing that complex tissue dynamics remain. These findings support multifunctional OCT as a novel preclinical tool for assessing anti-fibrotic therapies and suggest that, with refinement, this noninvasive imaging approach could inform clinical strategies for monitoring skin and potentially multi-organ involvement in systemic sclerosis and other fibrosing disorders.

## METHODS

### Study design

A total of 60 C57BL/6J male mice sourced from The Jackson Laboratory (6–8 weeks old) were utilized. Upon arrival, the mice underwent a period of acclimatization in a controlled facility, lasting at least 7 days, to minimize stress and allow them to adapt to the new environment. They were housed in groups to promote social behavior under strict environmental conditions with a 12-h light/dark cycle, temperature maintained at 22–24 °C, and humidity levels kept at 40%–60%. The mice had *ad libitum* access to a standard laboratory diet and water, ensuring their nutritional needs were met. Enrichment, including nesting materials and structures for hiding, was provided to support natural behaviors and enhance well-being. All procedures related to handling and treatment were designed to minimize stress and discomfort to the animals, adhering to the highest standards of animal welfare.

**FIG. 5.**
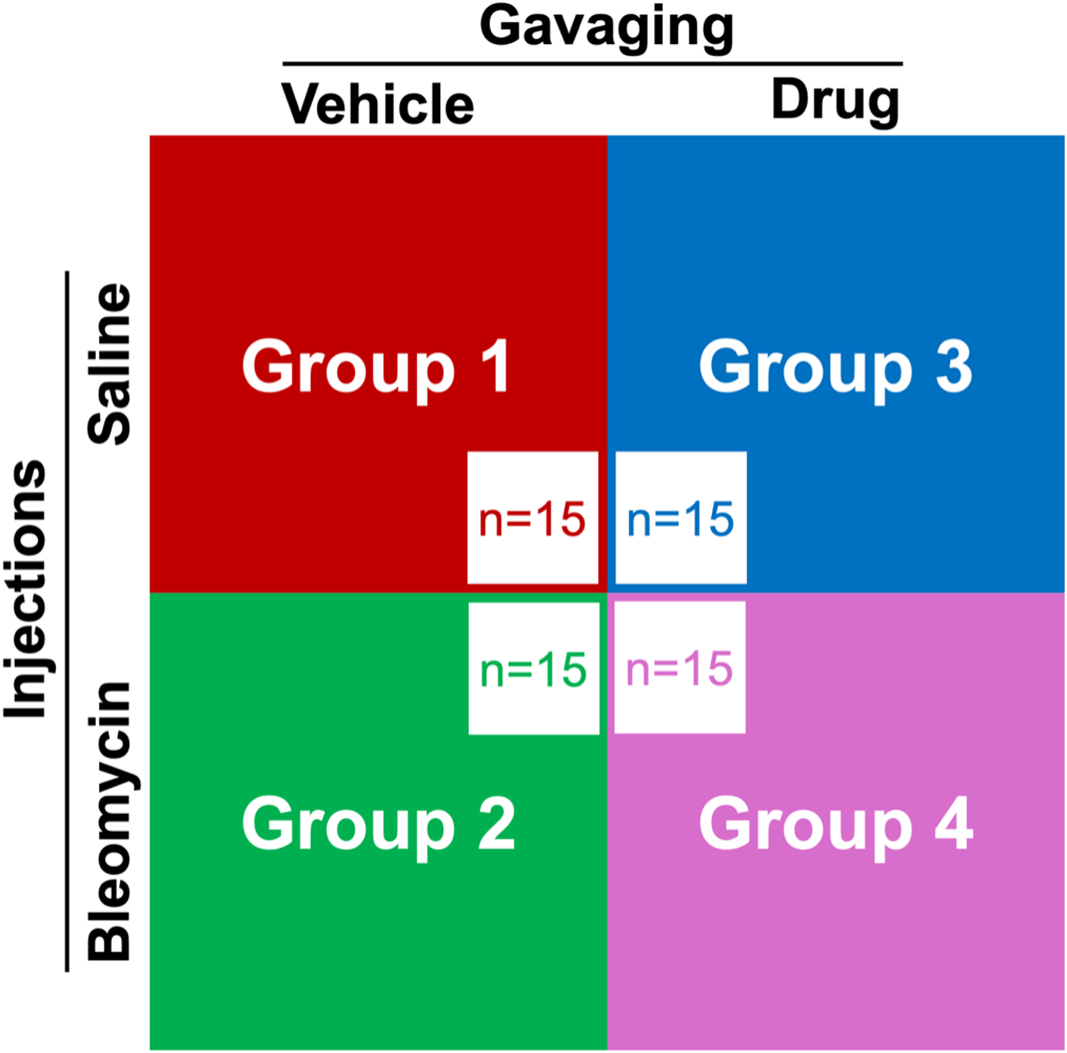
The experimental groups. Distribution of the C57BL/6J male mice (n = 60) across four groups. Group 1 (red) received saline injections and vehicle gavage. Group 2 (green) received bleomycin injections and vehicle gavage. Group 3 (blue) received saline injections and SB525334 gavage. Group 4 (purple) received bleomycin injections and SB525334 gavage. N = 15/group.

**FIG. 6.**
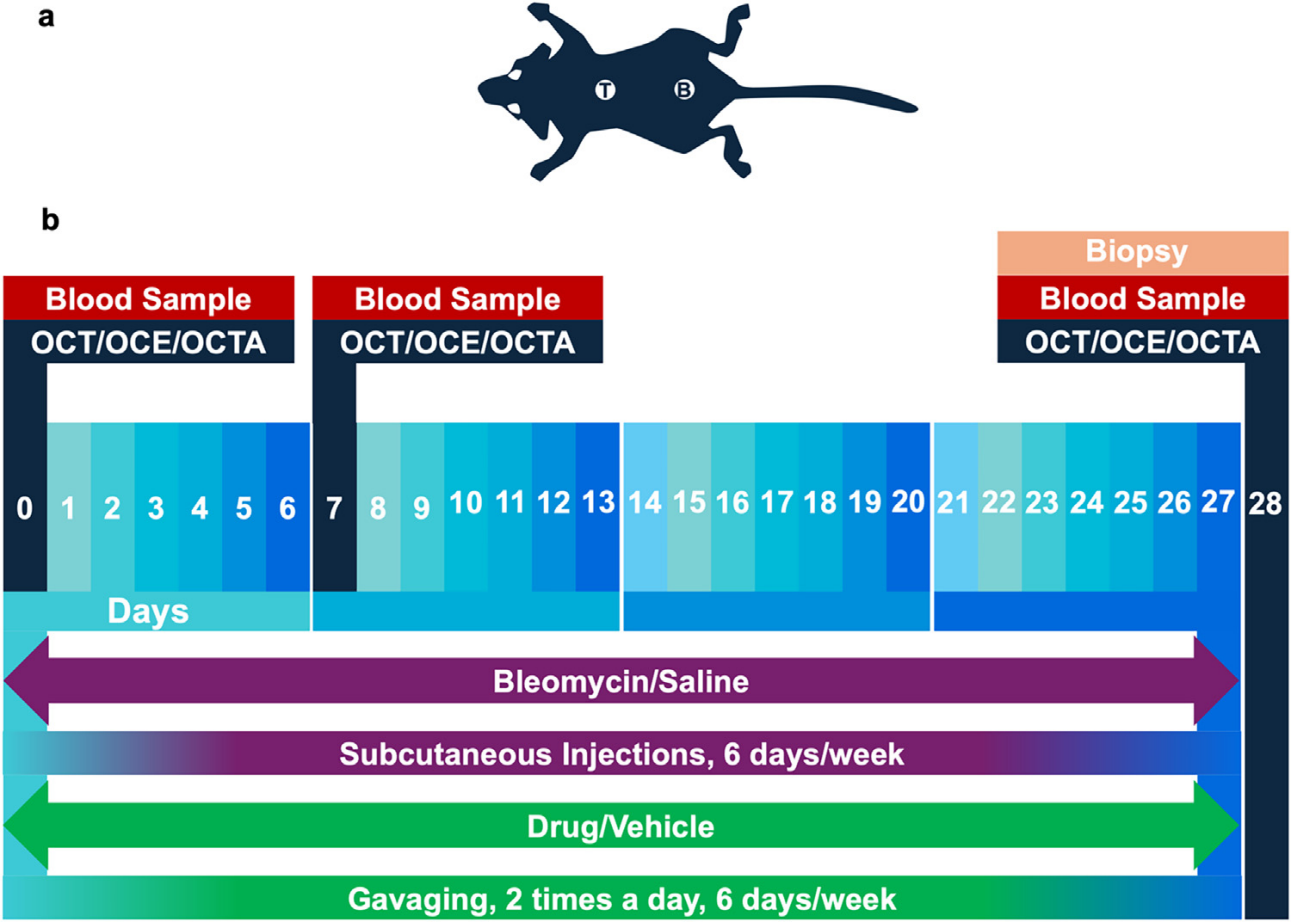
Timeline of experimental procedures. (a) Two locations of bleomycin/saline injections: the top location (T) and bottom location (B). (b) The experimental timeline over a four-week period, showing the schedule of subcutaneous bleomycin or saline injections and oral gavage of the SB525334 or vehicle, which were administered six days per week for four weeks. It also marks the points at which biopsies were performed, blood samples were taken, and OCT/OCE/OCTA imaging was conducted.

These mice underwent a bleomycin-induced skin fibrosis protocol, mirroring the pathological progression of skin fibrosis observed in SSc. Mice were divided into four groups to receive saline injections with intragastric gavage of the SB 525334 vehicle (group 1), bleomycin injections with vehicle gavages (group 2), saline injections with SB 525334 gavages (group 3), and bleomycin injections with SB 525334 gavages (group 4), as shown in Fig. 5. There were 15 mice in each of the four groups. Animals were subjected to daily injections of bleomycin or saline and intragastric gavage (6 days a week, gavage two times a day), the contents of which varied depending on the group. The study employed OCT/OCE/OCTA imaging at the baseline (day 0), one-week (day 7), and four-week (day 28) time periods (Fig. 6). A separate positive-control anti-fibrotic arm was not included because no universally accepted gold-standard agent exists for murine SSc, and adding such an arm would substantially increase animal use; efficacy was therefore evaluated relative to bleomycin + vehicle and saline controls.

Throughout imaging and data analysis, investigators were blinded to the treatment group assignments. Mice were assigned sequentially to the treatment groups as they became available for injections, without selection based on any particular characteristics, ensuring random allocation. Before the baseline and four-week end point imaging sessions, the hair on the back of the mice was removed using an electric shaver and a hair removal cream in such a way as to completely clear not only the injection and imaging points but also the area between and around these points. Hair removal was necessary to create an OCT/OCE/OCTA imaging window. During imaging, mice were anesthetized with isoflurane (5% for induction and 1%–3% for maintenance). Mice were then placed on a heated platform, maintaining a body temperature of 37 °C, ensuring their comfort and stability throughout the procedure.

### Ethical considerations

Group sizes were chosen based on prior analogous studies to detect meaningful differences across four arms, and all daily injections and gavage procedures were conducted by trained personnel under IACUC-approved protocols with adherence to the 3Rs (Replacement, Reduction, Refinement). Animals were monitored daily for weight, grooming, and distress. Predefined humane endpoints (>20% weight loss, severe ulceration, or persistent lethargy) triggered euthanasia, and peri-procedural analgesia (topical lidocaine) with heated-pad recovery was used. To mitigate stress, mice were habituated to handling, injections and gavage were performed under isoflurane when appropriate using soft feeding needles, the dosing order was randomized across groups, and no abnormal stress behaviors were observed in controls.

Animals were inspected once daily during acclimation and twice daily during the 28-day protocol (injection plus twice daily gavage). Clinical welfare was scored using a category-based rubric (appearance, posture/behavior, hydration, feces/urine, and procedure site), each rated 0–3, with predefined actions at score ≥2 in any category or ≥5 cumulative. Humane endpoints (pre-approved by the University of Houston IACUC) included ≥15% body-weight loss from baseline, persistent score ≥2 after supportive care, severe procedure-site complications, or moribund status. Animals meeting the criteria would be removed and euthanized. Refinements included pre-warmed gavage solutions, lubrication of gavage tips, rotating personnel to minimize handling stress, and alternating injection flanks/sites to limit local irritation. Across the cohort, there were no unplanned deaths, no animals met humane-end point criteria, and all animals completed the days 0, 7, and 28 imaging time points. Daily clinical scores remained in the normal–mild range, and no animal exceeded 10% weight loss from baseline during the study.

### Bleomycin and saline solution administration

Mice from groups 2 and 4 were injected with bleomycin to induce skin fibrosis. The dose of bleomycin was 20 *μ*g/mouse/day delivered subcutaneously six days a week for four weeks. Control animals in group 1 and group 3 were injected with saline in a similar manner and in an equivalent volume (100 *μ*l). For administering bleomycin and saline injections, we utilized 29G needles (Tenkaiwick, USA) attached to 500 *μ*l reusable glass syringes. This regimen aimed to closely mimic the pathological progression of fibrosis observed in SSc.^19,20,28,53^ Injections were carried out in two places: (1) in the lower back, closer to the caudal end of the mouse's body, (2) and in the upper back, closer to the cranial end of the mouse's body (Fig. 6). For standardization, the same two injection sites per mouse (“top” and “bottom”) were re-imaged at each time point by revisiting the positions marked relative to the injections.

### Drug and vehicle gavage procedures

Oral administration of SB 525334 and its vehicle was performed via intragastric gavage. Groups 3 and 4 received SB 525334 at a dose of 30 mg/kg, administered twice daily via oral gavage with a 6-h gap between daily doses. Groups 1 and 2 were administered vehicle in a volume equivalent to SB 525334 and following the same gavage procedures. This dosing regimen began concurrently with the start of the bleomycin or saline injections and continued throughout the four-week treatment period (6 days a week, similar to the injections). To ensure precise and safe administration, the gavage was performed using a 1 ml reusable glass syringe fitted with a 29G gavage needle. The size of the needle was specifically chosen to accommodate the small size of the mice while ensuring minimal discomfort during the procedure (Fig. 6). SB 525334 was given twice daily for 28 days to coincide with the entire bleomycin-challenge period and maintain continuous ALK-5 blockade, and published regimens vary (11–21 days), so this duration ensured full overlap without unnecessary animal burden. A single dose of 30 mg/kg/day was selected based on our prior study in this model and published PK/PD data for ALK5 inhibitors.^49^ A formal dose–response was not pursued to limit animal use and is outside the scope of this study. Mice were assigned at study start in a random manner to the four experimental groups. No allocation concealment or formal blinding was used during daily subcutaneous injections (bleomycin or saline) or oral gavage (SB-525334 or vehicle). The personnel administering treatments knew the group assignments. Imaging was acquired using the same preset parameters across all animals and time points. Quantitative outcomes (OCT thickness, OCE wave velocity, and OCTA vessel lumen width) were computed using pre-specified, deterministic analysis scripts applied uniformly to all datasets. These objective pipelines were used to minimize operator bias in the absence of formal blinding.

### OCT/OCE/OCTA setup and procedure

**FIG. 7.**
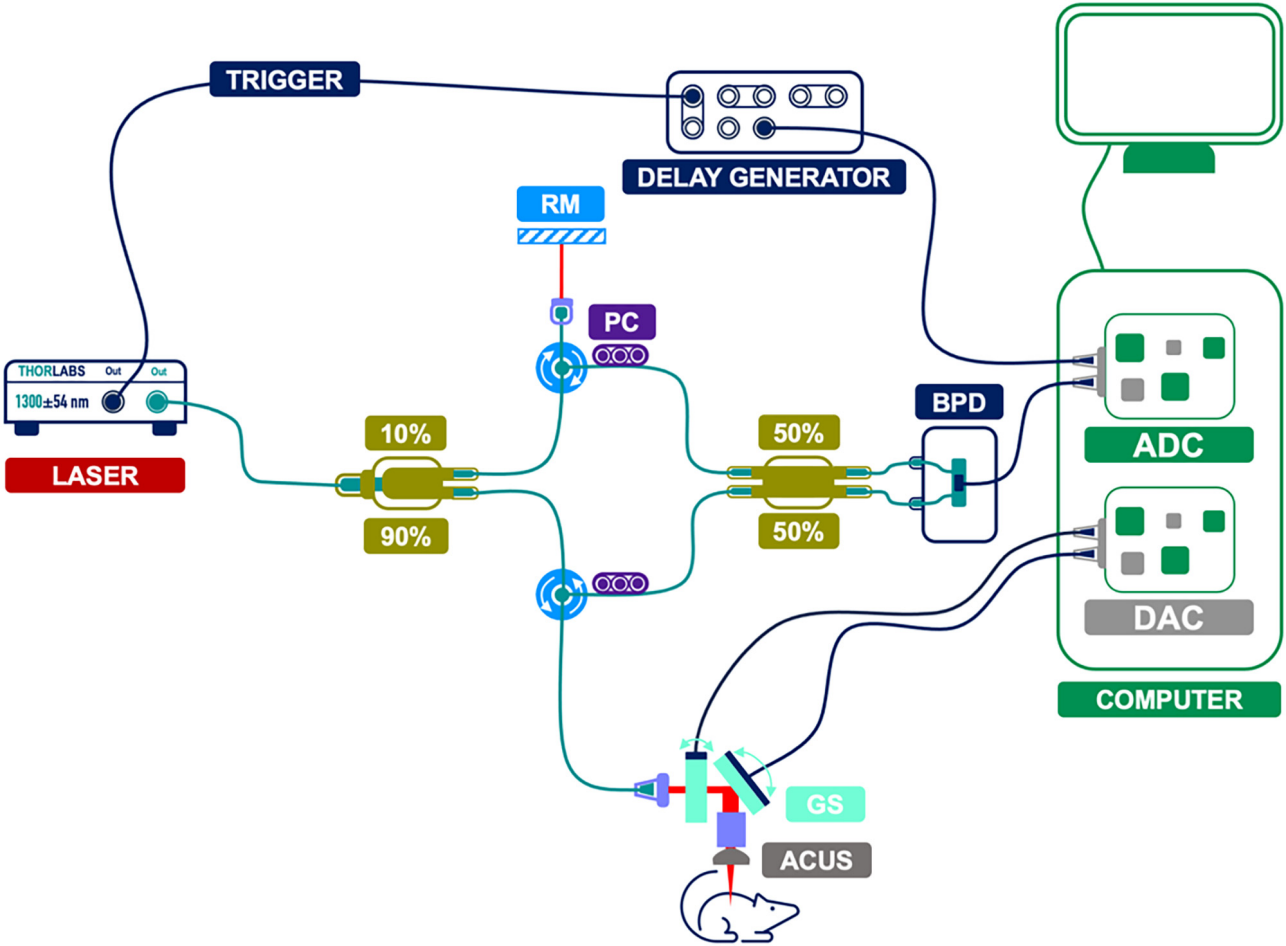
Optical coherence tomography (OCT) system schematic. This schematic diagram illustrates the configuration of the phase-sensitive swept source OCT system. It highlights the key components including the laser source, fiber couplers, reference mirror (RM), polarization controllers (PC), balanced photodetector (BPD), and the animal subject. The trigger and delay generator ensured precise timing for superior displacement stability, while the analog-to-digital converter (ADC) and digital-to-analog converter (DAC) facilitate data processing and scanner control and synchronization.

The OCT system was based on a microelectromechanical system vertical cavity surface emitting swept source laser (SL131091, Thorlabs Inc., USA) with a central wavelength of 1300 nm, a scan range of 108 nm, and a scan rate of 100 kHz. The OCT system had an axial resolution of 9 *μ*m and a lateral resolution of 27.8 *μ*m, both in air (Fig. 7). These resolutions are standard for murine skin OCT and sufficient to delineate the epidermis–dermis boundary used for thickness estimation; moreover, our analyses emphasize relative longitudinal and between-group changes, so any residual resolution-related bias would be uniform across groups and does not affect the comparative conclusions. The incident power on the sample was 16 mW. The laser sweep trigger was precisely aligned with the k-clock and digital acquisition for optimal displacement stability.^54^ With this setup, we achieved a stability of ∼41 pm. Imaging was performed on both sides of the injection site, and the full process of OCT, OCE, and OCTA imaging took approximately 5 min.

To image the skin structural properties, a three-dimensional OCT scan was performed over an area of 5.16 × 6.14 mm with 500 A-lines per B-scan and 500 B-scans per position. Five repeated measurements were taken for each B-scan for averaging to suppress noise and improve image quality.

OCE measurements were performed by exciting the skin with an air-coupled ultrasound (ACUS) transducer,^55,56^ which delivered a precise, non-contact stimulus. This method involves directing focused acoustic radiation force onto the skin surface for a transient period (less than 100 ms), which induces elastic-wave propagation. The ACUS approach was selected for its ability to provide tightly controlled mechanical excitation at 1, 5, and 10 kHz frequencies. Multi-frequency (1, 5, and 10 kHz) measurements were acquired by design to sample different depth-weighted layers, and the resulting frequency-dependent differences are expected and reflect viscoelastic heterogeneity rather than methodological error. The ACUS transducer used for OCE measurements had a resonant frequency of 1 MHz, with a focal distance of 20 mm and a diameter of 34 mm. The transducer had a hole in the center with a diameter of 10 mm for confocal imaging. Each excitation was performed with a single transient pulse at a 50% duty cycle and a train of multiple excitations at each excitation frequency.^56^ Each OCE scan was performed with the M–B-mode imaging paradigm.^36,50,57^ In each M-mode scan, there were 500 A-lines per M-mode image, with 500 M-mode images per scan. This technique, combined with the sub-nanometer displacement stability of phase-sensitive OCT,^58^ captured the elastic-wave propagation, offering a detailed insight into the skin's viscoelastic properties. For a more comprehensive assessment of the biomechanical properties of the tissue, scanning was carried out in two perpendicular directions, along and across the body's midline.

OCTA imaging utilized the OCT structural imaging data to isolate the microvasculature, as described later.

### Data processing

The data were processed to produce structural images, OCE visualizations, speed quantifications, and microvasculature maps. The processing of the collected imaging data was performed using MATLAB software (MathWorks, USA).

For measuring skin thickness, the five repeated scans at the same location were averaged. After averaging the five repeated scans at the same location, the outermost 100 images were removed from both the top and bottom of the stack to reduce potential edge artifacts. Intensity-based thresholding was used to track the top and bottom surfaces of the epidermis. Fifty A-lines were removed from each end of the B-scans. The pixel distances between the two surfaces were converted to real dimensions, assuming a skin refractive index of 1.376 (standard assumption^59,60^), and the skin thickness was quantified as the mean difference between the two surfaces for all the quantified A-lines per imaged location (a total of 600 000 A-lines per position). The skin of mice at later stages of the experiment, especially by the final imaging time point at 4 weeks, became heterogeneously pigmented, which significantly affected the assessment of skin thickness. Thus, regions with significant pigmentation were not included in the analysis. Pigmented areas were identified by intensity-based thresholding with manual confirmation and excluded uniformly across groups, comprising <10% of scanned regions of interest.

The biomechanical properties of the skin were assessed by tracking the propagation of the ACUS-induced mechanical wave in the skin with subsequent calculation of the group velocity. The group velocity was quantified separately for regions to the right and left of the stimulation since the mechanical waves were excited in the middle of the imaged field of view. The results were then averaged. This parameter was calculated separately for longitudinal (along the midline) and transverse (across the midline) measurements, after which the result for each location was averaged. Generally, a greater wave speed corresponds to a stiffer material.^36^

To reconstruct the microvasculature network from the 3D OCT data, a correlation mapping OCTA algorithm was employed with a kernel size of 5 pixels,^61^ enabling the detection of moving elements, i.e., blood cells in this case, between five repeated B-scans at each imaged position. A Hessian-filtering-based approach was used to highlight and emphasize the vessels. Subsequently, the vessel lumen width was manually quantified using QuPath software (University of Belfast, UK), with averages calculated for each location. Two investigators performed lumen measurements using the same predefined protocol because the criteria were deterministic and consistently applied, and formal inter-rater reliability was not calculated. To reduce motion, mice were anesthetized and imaged on a heated platform with less than 5-min scans. Processing followed published methods with the parameters listed here; the custom MATLAB scripts implementing these steps are available from the corresponding author upon reasonable request to facilitate replication.

### Sample collection and histopathology

Following the final imaging session on day 28, mice were humanely euthanized using isoflurane anesthesia. Punch biopsies (6 mm diameter) were taken from both injection sites of each animal. The tissues were meticulously collected and processed for histopathological examination. Masson's Trichrome staining for collagen fibers and dermal thickness, immunohistochemistry with mouse monoclonal α-SMA antibody for myofibroblast population, and hydroxyproline assay for total collagen content in the lesional skin were performed. Each skin biopsy sample was cut into two pieces. One half was fixed in formalin and processed into a paraffin-embedded block for Masson's Trichrome and α-smooth muscle actin (SMA) staining. The other half was immediately frozen and stored at −80 °C for hydroxyproline analysis. Briefly, 5 *μ*m paraffin sections were deparaffinized and rehydrated, and Masson's Trichrome staining was performed according to the manufacturer's protocol. Next, a mouse monoclonal α-SMA antibody was used for α-SMA positive myofibroblast staining. Briefly, tissues were deparaffinized and rehydrated. After antigen retrieval, mouse skin tissues were incubated with BLOCKALL blocking buffer for 10 min followed by mouse-on-mouse IgG blocking reagent incubated for 1 h. Mouse monoclonal α-SMA (Sigma-Aldrich; 1:×300 dilution) was added and incubated overnight at 4 °C. After 1 h of anti-mouse IgG-HRP secondary antibody incubation, DAB substrate was applied, followed by hematoxylin counterstain. The slides were covered with a permanent mounting medium and viewed under a light microscope. Representative images were taken for the analysis. The proportion of the positive fibroblasts with bright brown staining representing SMA of interest was counted in the four different field images from each sample from each animal. The α-SMA positive fibroblast number and the ratios of α-SMA positive fibroblast number to total fibroblast (spindled-shape cells exclusive of nerve and endothelial cells) number in each field were reported. Next, half of the 6 mm punch skin samples was used for the hydroxyproline assay. Briefly, skin tissues were homogenized in 100 *μ*l of water, and 100 *μ*l of hydrochloric acid (HCl, ∼12 M) was added, followed by hydrolysis at 120 °C for 3 h, according to the manufacturer's protocol. Materials and reagents are listed in Table III.

**TABLE III.** Reagents used for the histological and immunohistochemical part of the study.

Manufacturer	Product	Storage	Preparation
Sigma-Aldrich	Hydroxyproline assay kit (MAK008)	4°	Follow instruction
Sigma-Aldrich	Trichrome Stain (Masson) Kit (HT15)	R.T.	Follow instruction
Sigma-Aldrich	Mouse monoclonal α-SMA antibody (A5228)	−80°	1:×300 dilution, before use
Abcam	Mouse IgG isotype control (ab37355)	4°	1:×300 dilution, before use
Fisher Scientific	Hematoxylin	R.T.	Use asis
Vector Laboratories	M.O.M.^®^ (Mouse on Mouse) ImmPRESS^®^ HRP (Peroxidase) Polymer Kit (MP-2400)	4°	Follow instruction
Vector Laboratories	ImmPACT^®^ DAB Substrate Kit, Peroxidase (HRP) (SK-4105)	4°	Follow instruction
Vector Laboratories	Antigen Unmasking Solution, Citric Acid Based (H-3300-250)	4°	Follow instruction
Vector Laboratories	BLOXALL^®^ Endogenous Blocking Solution, Peroxidase and Alkaline Phosphatase (SP-6000-100)	4°	Follow instruction

### Statistical methods

We powered the study on the day-28 OCE elastic-wave velocity because it was the most sensitive imaging end point in our prior bleomycin model and has well-documented development of fibrosis at that stage. In that dataset, the between-group difference at day 28 (bleomycin vs saline) was 1.6 ± 0.3 vs 1.2 ± 0.2 m/s (n = 6 per group), yielding Hedges' g = 1.45. Using a two-sided α = 0.05 and 1 − β = 0.80 for a two-sample comparison, this effect size indicates that 8 animals per group are sufficient. We enrolled n = 15 per group to maintain ≥0.80 power while using nonparametric tests and to allow for attrition across our primary imaging endpoints. Under these assumptions, n = 15 per group provides ≥0.80 power to detect a minimum detectable difference of 0.26 m/s, which is ∼16% of the bleomycin-group mean at day 28.^49^ Since we did not assess data normality, we applied nonparametric tests for all statistical comparisons, namely, Wilcoxon Signed Rank tests for paired measurements and Mann–Whitney U tests for between-group comparisons. Because multiple comparisons were performed, p-values were adjusted post-hoc using the Bonferroni correction, and these adjusted values are reported in Tables I and II.

## SUPPLEMENTARY MATERIAL

See the supplementary material for Figs. 1–5 providing additional quantitative and visual documentation of the study outcomes. Figure S1 shows group velocity trajectories at the bottom site across all excitation settings (1, 5, and 10 kHz; single and multiple pushes), Fig. S2 shows the analogous trajectories at the top site, and Fig. S3 reports OCTA-derived vessel lumen widths by site. Figure S4 presents representative OCE space-time maps at 10 kHz single-push (bottom site), illustrating wave-speed increases with bleomycin and attenuation with SB 525334, and Fig. S5 shows structural OCT at the bottom site (days 0, 7, and 28), demonstrating bleomycin-induced thickening and partial mitigation with SB 525334. Group labels: G1—saline + vehicle, G2—bleomycin + vehicle, G3—saline + SB 525334, and G4—bleomycin + SB 525334. Statistical methods follow the main text.

## Data Availability

The data that support the findings of this study are available from the corresponding author upon reasonable request.
